# 

**Published:** 1898-03

**Authors:** 


					﻿Vol. XVIII.
MARCH, 1898.
No. 3.
IRHE
HOMEOPATHIC pHYSlClAH,
A Monthly Journal of Homoeopathic Materia Medica
and Clinical Medicine.
“ He it a freeman whom truth makes free, and all are slaves beside."
"Seek the Iruth: come whence it may, cost what it will."
EDITED AND PUBLISHED BT
WALTER M. JAMES, M. D.
PHILADELPHIA :
ZTSTo. 1231 LOCUST STZROEIET.
LONDON \
ALFRED HEATH & CO., Sole Agents fob Gbeat Britain,
114 Ebury Street, Eaton Square.
O“Tearly Subscription, $9.SO, invariably in advance. Single numbers, 9S cts.
Entered at the Philadelphia Poit-Oæce at Beoond-Claat Mail Matter.
				

